# Case report: Reconstruction of distal medial tibial epiphysis using iliac crest apophyseal autograft

**DOI:** 10.3389/fped.2022.950211

**Published:** 2022-08-12

**Authors:** Chengming Zhu, BaoJie Shi, Saroj Rai, Haobo Zhong, Xin Tang

**Affiliations:** ^1^Department of Orthopaedic, Liuzhou Workers Hospital/The Fourth Affiliated Hospital of Guangxi Medical University, Liuzhou, China; ^2^Department of General Surgery, Xiang'an Hospital of Xiamen University, Xiamen, China; ^3^Department of Orthopaedics and Trauma Surgery, Karama Medical Center, Dubai Investment Park Branch, Dubai, United Arab Emirates; ^4^Department of Orthopaedics, Huizhou First Hospital, Huizhou, China; ^5^Department of Orthopaedics, Union Hospital, Tongji Medical College, Huazhong University of Science and Technology, Wuhan, China

**Keywords:** physeal transplantation, reconstruction, Salter type VI physeal injury, medial ankle, children

## Abstract

**Background:**

Salter-Harris type VI physeal fracture is a rare injury. This case study aims to present a novel method for treating a rare entity of Salter-Harris type Salter-Harris VI physeal injury of the medial malleolus.

**Case presentation:**

A 6-year-old boy with Salter-Harris type VI physeal injury was successfully treated using the two-stage procedure. In the first stage, the patient was treated with intravenous antibiotics, a series of debridement and lavage followed by a skin graft that left a defect in the medial malleolus. In the second stage, an autogenous iliac crest apophyseal graft was transplanted to reconstruct the medial malleolus, and the ankle joint was stabilized by an external fixator. An additional anticipatory Langenskiold procedure was performed for the physeal bar resection. Although the complete radiological development of medial malleolus compared to the contralateral side was not evident at the last follow-up, the functional and cosmetic outcomes were satisfactory.

**Conclusion:**

The reconstruction of medial malleolus using an autologous iliac crest apophyseal graft and stabilization of the ankle joint with an external fixator is a novel reconstruction technique in treating Salter-Harris type VI physeal injury of the medial malleolus. This technique provides satisfactory functional and cosmetic outcomes in such a fracture pattern; however, a further clinical study using a larger sample size is warranted in order to find the definitive outcome of the technique.

## Introduction

Physeal injury of the distal tibia is the second most common among all physeal injuries having a tremendous risk of premature growth arrest resulting in angular deformity and leg length discrepancy (1). Initially, Salter and Harries in 1963 classified physeal injuries into five types (type I–V). Later, Peterson added type VI physeal injury to this classification as the loss of osteochondral piece along with the physis because the initial classification system did not classify such injury pattern (2). Type VI injury is usually caused by specific mechanisms, including a lawnmower, motor vehicle, boat propeller, etc., resulting in local injury and avulsion fragment (2). Despite being a rare type of physeal injury, this fracture has the highest rate of complications due to an osteochondral defect in growing children.

A case of a 6-year-old boy with an open Salter-Harris type VI physeal injury and a complete loss of medial malleolus was presented in this case report. The deformity was treated using the two-stage procedure. In the first stage, the patient was treated with intravenous antibiotics, a series of debridement and lavage followed by a skin graft that left a defect in the medial malleolus. In the second stage, an autogenous iliac crest apophyseal graft was transplanted to reconstruct the medial malleolus, and the ankle joint was stabilized by an external fixator. An additional anticipatory Langenskiold procedure was performed for the physeal bar resection. Finally, this patient achieved a satisfactory functional and cosmetic outcome. This case report was approved by the ethical review board of the author's institute, and written informed consent was obtained from the patient's legal guardian.

## Case report

A 6-year-old boy was brought to the orthopedic emergency department following a motor vehicle accident (MVA) with an injury to his right ankle. He complained of pain on the right side of his foot and difficulty in walking, with obvious deformity of the injured ankle. The clinical and radiological examination revealed the loss of overlying skin and an osteochondral fragment from the medial malleolus. The condition was diagnosed as the Salter-Harris type VI physeal injury of the medial malleolus of the right ankle.

**Figure 1:**
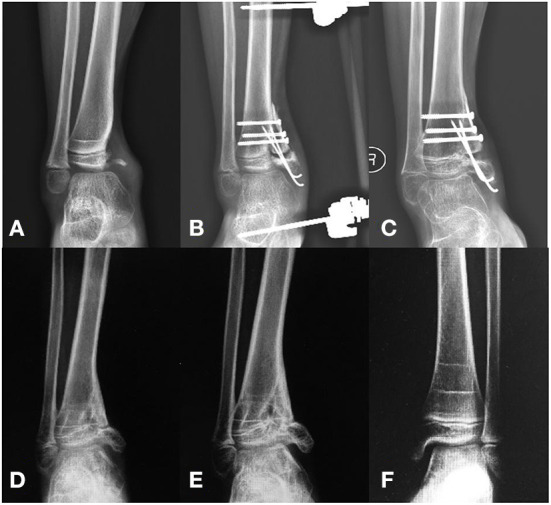
Anteroposterior X-ray view of a 7.5 years old boy shows the ankle joint in varus at 20 months after the Salter type VI physeal injury **(A)**. Post-operative X-ray shows a wedge-shaped iliac apophyseal bone autograft **(B)**. Five months post-operative X-ray shows the united iliac crest apophyseal autograft **(C)**. A physeal bar on the distal medial tibia was observed 20 months after reconstructive surgery **(D)**. On 36 months follow up after reconstructive surgery, the reconstructed medial malleolus **(E)** compared with the contralateral side **(F)**.

The patient has been treated with a two-stage procedure. In the first stage in emergency department, the wound was meticulously washed, debrided, and kept in a vacuum sealing drainage for 5 days. Intravenous antibiotics were administered as a part of the management of open fractures. The wound bed was prepared for granulation tissue formation. After the formation of healthy granulation tissue, the split-thickness skin graft was grafted over it with a cast for immobilization by injury surgeons. The boy was discharged from the hospital 7 days after skin grafting and requested follow-up at the outpatient clinic. However, the boy lost the follow-up. At 20 months after the primary surgery, the boy again appeared in the hospital complaining of significant ankle pain, deformity, and abnormal gait. On examination, there was an obvious varus deformity of the ankle joint. Radiographic examination revealed the medial malleolus defect with cavovarus deformity of the right ankle (Figure 1A).

**Figure 2:**
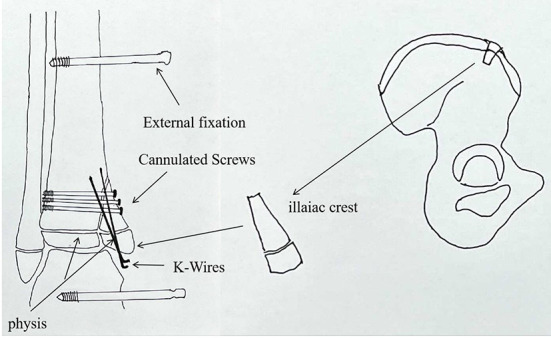
An illustration of the surgical plan using an iliac crest apophyseal autograft. The graft is held together by screws and k-wires, and the ankle is held by the external fixator.

**Figure 3:**
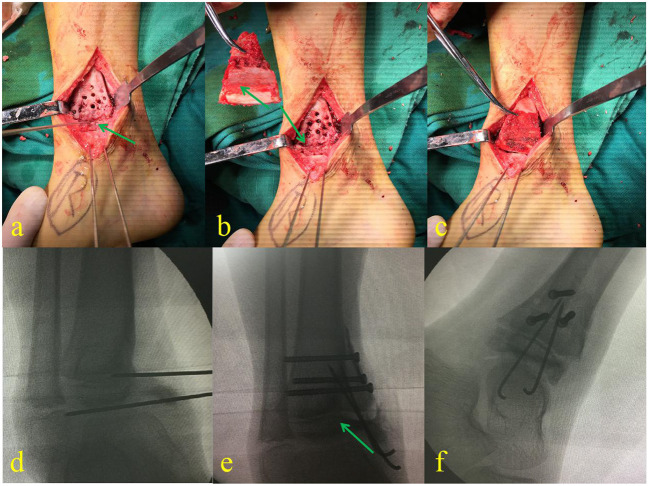
Debridement and location of the distal tibial physis with a K-wire **(a)**. Harvested iliac crest apophyseal autograft, ready to insert in an appropriate place **(b)**. The cartilage of the iliac crest autograft was positioned to meet the medial aspect of the talus **(c)**. **(d–f)** Are the fluoroscopic images taken during the surgery.

In the second stage, the boy was treated with the reconstruction of the medial malleolus using an autogenous iliac crest apophyseal graft (Figure 2) by pediatric orthopedic surgeons. In this procedure, the graft was placed on the medial malleolus in such a way that the physeal line of the iliac crest graft was parallel to the physeal line of the distal tibia (Figure 3). The graft was then held by 4 mm cannulated screws (Asnis III stainless steel screws, Stryker, France) and 1.5 mm Kirschner wires (K-wires) (Figure 1B). The ankle joint was then stabilized with an external fixator (Trauson, ChangZhou, China) without the cast, in order to provide longitudinal traction to avoid unnecessary pressure on the graft and correct the cavovarus deformity.

**Figure 4:**
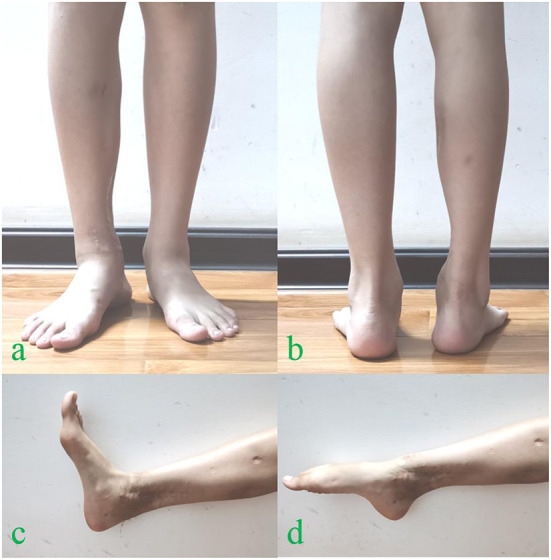
At 36 months follow-up after reconstructive surgery, the apparent deformity was not evident **(a,b)**, the functional and cosmetic outcomes of the operated ankle **(c,d)**.

The external fixator was removed after 3 months when the radiological evidence of graft union was present (Figure 1C), and the remaining implants were removed after 12 months following the second surgery. After 20 months following the reconstruction, a physeal bar on the distal medial tibia appeared on the radiograph (Figure 1D), which was excised using the Langenskiold procedure. Despite such an effort, the reconstructed medial malleolus did not develop as expected compared to the contralateral side (Figures 1E,F). There was still a residual varus deformity of the ankle joint; however, no leg length discrepancy was evident 36 months after the second operation. Despite the residual deformity, functional and cosmetic evaluation score was satisfactory, and further supra malleolar osteotomy was not required (Figure 4).

## Discussion

Physeal injury is common in children that account for ~15–18% of all pediatric fractures (3). Anatomic reduction is the mainstay of treatment (4–6). The most widely used classification for physeal injury was suggested by Salter and Harris in 1963. However, it does not describe certain physeal injuries. Salter-Harris type VI injury is rare in which an osteochondral fragment and a portion of physis are lost, bringing significant complications, including angular deformity and growth arrest resulting in leg length discrepancy. Only a few cases have been reported in the literature regarding this fracture pattern until now (7–10).

Peterson and Jacobsen (10) described 4 different treatment options for Salter-Harris type VI physeal injury based on the degree of bone loss and the presence of the bone defect. The authors suggested an application of fat tissue or iliac apophyseal cartilage in acute injury. They suggested using either an iliac crest bone graft or corrective osteotomy, or both in case of delayed injury (10). A prophylactic Langenskiold procedure was recommended by Foster et al. (11), and they reported satisfactory outcomes in both the acute and delayed stages. A correct autologous bone transplant or graft is mandatory for the success of this technique (12). Abbo et al. (9) recommended the utilization of prophylactic physiolysis with the interposition of fat in an acute stage in order to avoid repeated corrective osteotomies in the growing bone.

It is challenging to reconstruct Salter-Harris type VI physeal injuries in one stage because an open injury has a high risk of complications such as infection. To avoid such complications, we used a two-stage technique. In the first stage, only wound care was performed with intravenous administration of antibiotics, wound lavage, and debridement. The wound bed was prepared for the healthy granulation tissue to grow so that the skin graft could be possible later. Another issue is that even if the soft tissue condition is relatively good in the acute stage, it's challenging to perform a free flap and malleolar reconstruction simultaneously. To avoid such complications, reconstruction is usually performed in the second stage after the complete healing of the wound with no evidence of the infection.

The age of the patient and degree of bony loss are crucial factors for developing deformity and growth arrest. For optimum outcomes, the surgeon should always consider these factors before planning the reconstructive surgery. Our patients' age was only 7.5 years at the time of reconstructive surgery, so the risk of post-operative deformity was almost inevitable. The medial malleolus was reconstructed with an iliac apophyseal autograft covering the medial aspect of the talus. The physeal line of the autograft was placed parallel to the physeal line of the distal tibia with the hope that the autograft would heal appropriately and allow for potential growth.

In contrast to Abbo et al. (9), an external fixator was used to avoid the contracture and varus collapse of the ankle joint. The external fixator also reduces the longitudinal pressure on the newly reconstructed medial malleolus. It was removed when there was radiological evidence of graft union at 3 months post-operatively.

At the last follow-up, the age of the patient was 10.5 years. No length discrepancy in our case might be because of the early excision of the bony bar, which was <20% of the distal tibia physeal scope. Although the complete union was evident at the last follow-up, the medial malleolus did not develop as on the contralateral side. However, the external deformity was not apparent, and the patient had satisfactory cosmetic and functional outcomes. Epiphysiodesis is a valuable option to prevent further deformity.

The reconstruction of medial malleolus using an autologous iliac crest apophyseal graft and stabilization of the ankle joint with an external fixator is a novel reconstruction technique in treating Salter-Harris type VI physeal injury of the medial malleolus. Our patient reported satisfactory functional and cosmetic outcomes at the final follow-up; however, a further clinical study using a larger sample size is warranted in order to find the definitive outcome of the technique.

## Data availability statement

The original contributions presented in the study are included in the article/supplementary material, further inquiries can be directed to the corresponding author.

## Ethics statement

The studies involving human participants were reviewed and approved by the Ethics Committee of Tongji Medical College, Huazhong University of Science and Technology (IORG No: IORG0003571). Written informed consent to participate in this study was provided by the participants' legal guardian/next of kin. Written informed consent was obtained from the individual(s), and minor(s)' legal guardian/next of kin, for the publication of any potentially identifiable images or data included in this article.

## Author contributions

BS were involved in data collection and follow-up assessments. XT, SR, and HZ were responsible for literature search, study design, and finalized the manuscript. CZ drafted the manuscript. All authors contributed to the article and approved the submitted version.

## Conflict of interest

The authors declare that the research was conducted in the absence of any commercial or financial relationships that could be construed as a potential conflict of interest.

## Publisher's note

All claims expressed in this article are solely those of the authors and do not necessarily represent those of their affiliated organizations, or those of the publisher, the editors and the reviewers. Any product that may be evaluated in this article, or claim that may be made by its manufacturer, is not guaranteed or endorsed by the publisher.
