# Corrigendum: Case report: Reconstruction of distal medial tibial epiphysis using iliac crest apophyseal autograft

**DOI:** 10.3389/fped.2023.1165785

**Published:** 2023-03-02

**Authors:** Chengming Zhu, BaoJie Shi, Saroj Rai, Haobo Zhong, Xin Tang

**Affiliations:** ^1^ Department of Orthopaedic, Liuzhou Workers Hospital/The Fourth Aliated Hospital of Guangxi Medical University, Liuzhou, China; ^2^ Department of General Surgery, Xiang’an Hospital of Xiamen University, Xiamen, China; ^3^ Department of Orthopaedics and Trauma Surgery, Karama Medical Center, Dubai Investment Park Branch, Dubai, United Arab Emirates; ^4^ Department of Orthopaedics, Huizhou First Hospital, Huizhou, China; ^5^ Department of Orthopaedics, Union Hospital, Tongji Medical College, Huazhong University of Science and Technology, Wuhan, China

**Keywords:** physeal transplantation, reconstruction, salter-Harris type VI physeal injury, medial ankle, children

A Corrigendum on Case report: Reconstruction of distal medial tibial epiphysis using iliac crest apophyseal autograft Zhu C, Shi B, Rai S, Zhong H and Tang X. (2022) Front. Pediatr. 10:950211. doi: 10.3389/fped.2022.950211

In the published article, there was an error in affiliation 5. Instead of “^5^Department of Orthopaedics, Tongji Hospital, Tongji Medical College, Huazhong University of Science and Technology, Wuhan, China”, it should be “^5^Department of Orthopaedics, Union Hospital, Tongji Medical College, Huazhong University of Science and Technology, Wuhan, China”.

The authors apologize for this error and state that this does not change the scientific conclusions of the article in any way. The original article has been updated.

